# Gait Recovery with an Overground Powered Exoskeleton: A Randomized Controlled Trial on Subacute Stroke Subjects

**DOI:** 10.3390/brainsci11010104

**Published:** 2021-01-14

**Authors:** Franco Molteni, Eleonora Guanziroli, Michela Goffredo, Rocco Salvatore Calabrò, Sanaz Pournajaf, Marina Gaffuri, Giulio Gasperini, Serena Filoni, Silvano Baratta, Daniele Galafate, Domenica Le Pera, Placido Bramanti, Marco Franceschini, on behalf of Italian Eksogait Study Group

**Affiliations:** 1 Villa Beretta Rehabilitation Center, Valduce Hospital, Costa Masnaga, 23845 Lecco, Italy; fmolteni@valduce.it (F.M.); eleonora.guanziroli@gmail.com (E.G.); mgaffuri@valduce.it (M.G.); ggasperini@valduce.it (G.G.); 2 Neurorehabilitation Research Laboratory, IRCCS San Raffaele Pisana, 00163 Rome, Italy; sanaz.pournajaf@sanraffaele.it (S.P.); daniele.galafate@sanraffaele.it (D.G.); domenica.lepera@sanraffaele.it (D.L.P.); marco.franceschini@sanraffaele.it (M.F.); 3 Neurorobotic Rehabilitation, IRCCS Centro Neurolesi “Bonino-Pulejo”, 98124 Messina, Italy; salbro77@tiscali.it (R.S.C.); bramanti.dino@gmail.com (P.B.); 4 Fondazione Centri di Riabilitazione Padre Pio Onlus, 71013 Foggia, Italy; serena.diba@gmail.com; 5 SCRIN Trevi Dipartimento di Riabilitazione USL Umbria 2, 06039 Perugia, Italy; silvano.baratta@uslumbria2.it; 6 Department of Human Sciences and Promotion of the Quality of Life, San Raffaele University, 00166 Rome, Italy

**Keywords:** stroke, robot-assisted gait training, exoskeleton device, neurologic gait disorders, rehabilitation

## Abstract

Background: Overground Robot-Assisted Gait Training (o-RAGT) provides intensive gait rehabilitation. This study investigated the efficacy of o-RAGT in subacute stroke subjects, compared to conventional gait training. Methods: A multicenter randomized controlled trial was conducted on 75 subacute stroke subjects (38 in the Experimental Group (EG) and 37 in the Control Group (CG)). Both groups received 15 sessions of gait training (5 sessions/week for 60 min) and daily conventional rehabilitation. The subjects were assessed at the beginning (T1) and end (T2) of the training period with the primary outcome of a 6 Minutes Walking Test (6MWT), the Modified Ashworth Scale of the Affected lower Limb (MAS-AL), the Motricity Index of the Affected lower Limb (MI-AL), the Trunk Control Test (TCT), Functional Ambulation Classification (FAC), a 10 Meters Walking Test (10MWT), the modified Barthel Index (mBI), and the Walking Handicap Scale (WHS). Results: The 6MWT increased in both groups, which was confirmed by both frequentist and Bayesian analyses. Similar outcomes were registered in the MI-AL, 10MWT, mBI, and MAS-AL. The FAC and WHS showed a significant number of subjects improving in functional and community ambulation in both groups at T2. Conclusions: The clinical effects of o-RAGT were similar to conventional gait training in subacute stroke subjects. The results obtained in this study are encouraging and suggest future clinical trials on the topic.

## 1. Introduction

Approximately 65% of stroke survivors suffer from sensory, motor, and cognitive impairment, as well as a reduced ability to perform self-care and participate in social and community activities, with a negative impact on their autonomy and quality of life [1,2]. Overground Robot-Assisted Gait Training (o-RAGT) is a recent cutting-edge neurorehabilitation treatment based on the Wearable Powered Exoskeleton (WPE) which helps a subject to walk over a hard surface in a clinical environment [3]. The WPE assists the movement of the lower limbs through a preprogrammed physiological gait pattern while the steps are triggered by the therapist or by the user with a weight shift-based trigger. High-tech WPEs are able to emulate overground human neuromotor control of locomotion, and they can provide early, intensive, and specific gait training. The o-RAGT could represent an innovative solution, compared with conventional gait training, since it provides intensive overground gait training even in subjects who are not able to maintain an upright position. In severely impaired walking subjects, it induces a coordinated, multisensory motor control stimulation and provides proprioceptive input to subjects during limb loading—which is associated with visual motor control—in order to properly navigate an exercise setting [4]. These features make the WPE a device that engages the subject and stimulates the mechanisms of visual exploration and motor coordination. Since these mechanisms are crucial for the restoration of motor control, o-RAGT could be considered a rehabilitation treatment which generates a more complex and controlled multisensory stimulation of the subject and which may modify the plasticity of neural connections through the experience of movement [5]. Moreover, the WPE not only moves the lower limb with a physiological gait pattern, but it also exhibits close cognitive and physical interaction with the subject [6]. The role of human–robot cognitive interaction in rehabilitation is to allow the patient to understand the potential of the robot, fully rely on the robot, and control the robot’s movements (when the steps are triggered by the patient with a weight shift-based trigger). For these reasons, the analysis of the usability and acceptance of o-RAGT is important to assess this human–robot cognitive interaction.

The literature on robotic gait training in neurological subjects includes a consistent number of Randomized Controlled Trials (RCTs) and systematic reviews [7,8,9]. Although well-established clinical outcomes have been obtained with treadmill-based robots [10,11,12], few early studies on o-RAGT have been published, with encouraging preliminary results obtained from limited sample sizes [3,4]. Specifically, the effects of o-RAGT in chronic stroke subjects were studied by a number of small RCTs that reported promising neurophysiological effects [13] and improvements in functional status [14], spatiotemporal gait parameters [15,16], and cardiopulmonary metabolic energy efficiency during walking [15]. On the other hand, subacute stroke subjects have received limited attention [17], even though rehabilitation in this phase has been identified as an important prognostic factor for a positive clinical outcome, and early verticalization and gait training seem to enhance beneficial neuroplasticity and cognitive and functional recovery. Encouraging preliminary results on o-RAGT in subacute stroke subjects were found in terms of the recovery of independent walking [18,19] and locomotor functions [20,21,22]. However, the WPEs are increasingly being used in clinical practice, and their efficacy needs to be studied. The purpose of this study was to assess the efficacy of employing an overground WPE for gait training in subacute stroke subjects, investigating the clinical effects compared to conventional gait training.

## 2. Materials and Methods

A multicenter, randomized, per-protocol controlled trial (NCT03395717) was carried out with a group of subjects who satisfied the following criteria: a first cerebral stroke (diagnosis confirmed by computed tomography scan or magnetic resonance imaging) with a Total Anterior Circulation Infarct (TACI), Partial Anterior Circulation Infarct (PACI), or Lacunar Anterior Circulation Infarct (LACI); a delay since the stroke ≤6 months (subacute phase); an age between 18–80 years; the ability to fit into the WPE (height between 150–190 cm and weight <100 kg); the ability to maintain an upright standing position for at least 60 s; and the ability to give written consent and comply with the study procedures. The exclusion criteria included persistent contractures of the joints that might limit the range of motion during the gait with the WPE, medical issues (e.g., orthopedic injuries, pain, severe osteoporosis, or severe spasticity), a history of significant problems with skin breakdown or current skin breakdown, inability to understand and comply with the study procedures, pregnancy, and untreated deep vein thrombosis (subjects who had been diagnosed with deep vein thrombosis and who were in the process of developing a correct and stable dosage of the pharmacological treatment).

Written informed consent was signed by each subject. Ethical approval of the treatment and evaluation protocol was guaranteed by the ethics committee of the main research hospital (IRCCS San Raffaele Pisana, Rome, Italy; date: 18 November 2015; code number: RP09/15). All centers had a standardized methodology for both the assessment and treatment processes. Specifically, before the beginning of the study, all clinicians and physical therapists involved in the study participated in meetings for defining a common and standardized approach for administering the clinical scales and treatments.

### 2.1. Treatments and Randomization Procedure

All subjects conducted daily conventional rehabilitation (120 min of rehabilitative intervention six days a week) consisting of physical therapy (e.g., upper limb functional task practice, upper limb muscle strengthening, and exercises for improving balance), speech therapy, and occupational therapy. Moreover, the subjects underwent gait training with a specific protocol, depending on the group assignment: (1) subjects who underwent gait training with the WPE (i.e., the Experimental Group (EG)) and (2) subjects who underwent conventional gait training (i.e., the Control Group (CG)). The start of the gait training was decided by the clinicians when the subject’s clinical status was suitable for such therapy. The duration of both the conventional gait training and the o-RAGT was the same (60 min). However, the intensity (i.e., the number of steps conducted by the patient in each session of treatment) was different; the o-RAGT was a more intensive form of gait training than the conventional one. This was because the WPE actively supported the patient in executing a physiological and timed gait.

All patients were sequentially assigned to either the EG or CG subsequent to enrollment in the study by using an envelope randomization technique. The randomization was performed in a 1:1 equal allocation ratio. The opaque, sealed, and sequentially numbered randomization envelopes were mixed and distributed to the research hospitals by the main research center. A randomization envelope was opened at the time of recruitment for each patient who met the inclusion criteria.

#### 2.1.1. The Overground Robot-Assisted Gait Training

The EG included subjects who underwent 15 sessions (5 days/week for 3 weeks) of gait rehabilitation with a WPE (Ekso™, Ekso Bionics, Richmond, CA, USA) which allowed them to stand up, sit, and walk overground. The subjects could use canes, crutches, or a walker during the o-RAGT. According to previous studies on the same device [21,22], the following WPE settings were used: ProStep Plus™ (each step was triggered by the subject’s transfer load from one leg to the other) and Bilateral Max Assist (the amount of power contribution to the legs during walking was totally provided by the robot). The WPE gait kinematic parameters were fine-tuned using surface electromyography, as detailed by Gandolla et al. [23]. Each session lasted 60 min (including approximately 15 min of the WPE donning and doffing) and consisted of the following motor tasks: sit-to-stand; re-adaptation to verticalization and awareness of the position; training of proprioception, balance, and load shifting while standing and during the gait; stepping and relearning of the correct gait pattern; and gradual adaptation to speed and resistance during walking.

#### 2.1.2. The Conventional Gait Training

The CG included subjects who underwent 15 sessions (5 days/week for 3 weeks) of conventional training consisting of the following tasks: lower limb stretching; lower limb muscle strengthening; static and dynamic balance exercises, proprioception, and trunk control exercise; a gait at the parallel bars or in clinical open spaces performed both with and without assistive devices (canes, crutches, or a walker); and training of climbing up and down stairs. Each session lasted 60 min.

### 2.2. Recruitment

**Figure 1:**
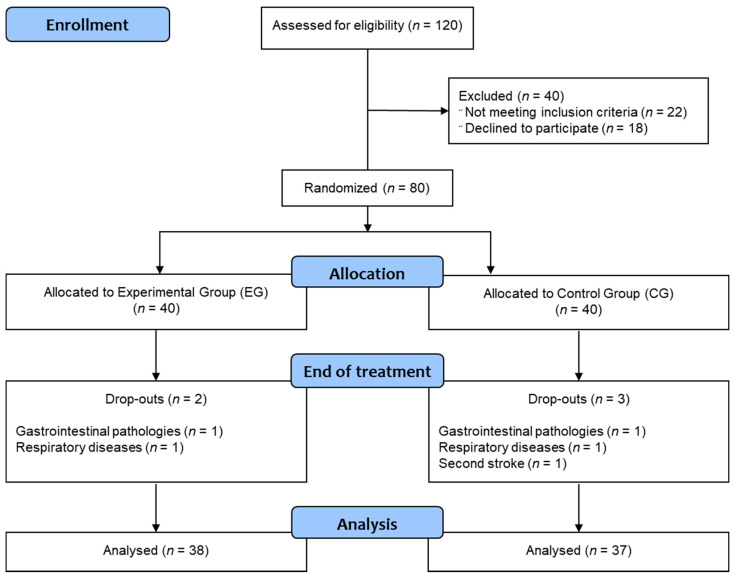
Flowchart.

A total of 120 subacute stroke subjects were recruited from March 2016 to April 2019 in five Italian rehabilitation hospitals. Ninety-eight subjects met the inclusion criteria, and 80 agreed to participate in the study, with 40 in the EG and 40 in the CG. Five subjects (2 in the EG and 3 in the CG) dropped out due to medical issues which were not related to the training (2 for gastrointestinal pathologies, 2 for respiratory diseases, and 1 for a second stroke). Finally, 75 subjects (38 subjects in the EG (62.13 ± 8.75 years old, 55% male) and 37 subjects in the CG (68.24 ± 8.58 years old, 49% male) conducted the rehabilitation treatment as detailed in the flowchart (Figure 1).

### 2.3. The Clinical Outcomes

The subjects were assessed at the beginning (T1) and end (T2) of the 3-week-long period of treatment with a set of clinical scales based on the International Classification of Functioning, disability, and health (ICF). The primary outcome of the study was the distance (m) covered over a time of 6 min (i.e., the 6 Minutes Walking Test (6MWT)).

For the body function and structure ICF domain, the Modified Ashworth Scale of The Affected lower Limb (MAS-AL) was employed to evaluate the muscle spasticity of the hip adductor, knee extensor, and ankle plantar flexor [24], and the Motricity Index of the Affected lower Limb (MI-AL) was employed to measure limb muscle strength [25].

For the activity ICF domain, the Trunk Control Test (TCT) was employed to assess the trunk control [26], the Functional Ambulation Classification (FAC) was employed to evaluate the basic motor skills necessary for functional ambulation [27], the 10 Meters Walking Test (10MWT) was employed to evaluate the maximum walking speed over a short distance [28], the 6MWT was employed as a sub-maximal test of aerobic capacity or endurance [29], and the modified Barthel Index (mBI) was employed to assess independence in activities of daily living [30].

For the participation ICF domain, the Walking Handicap Scale (WHS) was employed to assess the community ambulation [31].

### 2.4. Usability and Acceptance

At the end of the o-RAGT, the subjects in the EG were asked to answer a questionnaire about the acceptance of the o-RAGT [32]. The items in the questionnaire were related to the perceived degree of some aspects of robotic therapy, namely comfort, presence of pain, presence of fatigue, enjoyment, advantages, desire to continue, and suggested to anyone. Each subject was asked to assign a score (0–7) to each of the seven items as follows: disagree strongly (0), moderately (1), somewhat (2), or a little (3) and agree a little (4), somewhat (5), moderately (6), or strongly (7).

### 2.5. Data Analysis and Statistical Analysis

The data were imported into statistical software for analysis (JASP Team (2020). JASP (Version 0.14.1, Amsterdam, The Netherlands) (Computer software)) which was used for all statistical analyses. Descriptive statistics were calculated to illustrate clinical and demographic characteristics. The mean and standard deviation, or the frequency with relative percentages, were computed for the continuous and categorical variables, respectively. Normality was assessed by the Kolgomorov–Smirnov test. Baseline differences between the two groups were studied with an independent *t*-test or a chi-squared test for continuous or categorical variables, respectively. Both frequentist and Bayesian analyses were computed. For the frequentist analyses, repeated measures ANalysis Of VAriance (ANOVA) was computed to evaluate the effects between groups after the intervention. When a significant difference between groups was detected (statistical significance was set at an a priori alpha level of 0.05), post hoc analysis was run to compare the two treatments (Bonferroni corrections were applied when needed). These frequentist analyses were supplemented with Bayesian analyses because Bayesian analyses do not assume large samples, and typically smaller sample sizes can be analyzed without losing power while retaining precision [33,34].

The Bayesian repeated measures ANOVA was based on comparisons between models that included the factors of the group and time against a null model, which only includes the effects of the age and subject. The ratio of the likelihood of the data in the null model and in the models that included a specific factor (group/time) was quantified as a Bayes Factor (BF_01_). BF_01_ expressed relative support for one model over another model, given the data. The classification scheme [35] proposed a series of labels, for which specific BF_01_ values can be considered no evidence (BF_01_ = 1), anecdotal (1 < BF_01_ ≤ 3), moderate (3 < BF_01_ ≤ 10), strong (10 < BF_01_ ≤ 30), very strong (30 < BF_01_ ≤ 100), or extreme (BF_01_ > 100) relative evidence for the hypothesis of a difference between groups. The likelihood for the data in all models with a specific factor (group/time), compared to all models without it, was further quantified as BF_inclusion_. The effects were followed up by using the Bayesian equivalent of independent, one-sided *t*-tests.

A contingency table and chi-square hypothesis test of independence were used for comparing the number of subjects in the EG and CG reaching the Minimal Clinically Important Difference (MCID) threshold of improvement or no MCID improvement in the 6MWT. A chi-squared test of independence was utilized to study the intergroup differences for the categorical variables (MAS-AL, FAC, and WHS) at T1 and T2.

A secondary descriptive analysis was conducted by dividing the subjects with a criterion based on the walking ability at the baseline. Specifically, the subjects were divided into those unable to walk, defined as FAC = 0 (unable walking subject subgroup), and those able to do so, defined as FAC > 0 (able walking subject subgroup).

## 3. Results

Seventy-five subjects (38 subjects in the EG and 37 in the CG) conducted the rehabilitation treatments detailed in the previous section. No adverse events were evident during the gait training. Subjects in the EG tolerated the robotic treatment well and perceived the o-RAGT positively. The demographic and clinical characteristics of the sample are depicted in Table 1. At the baseline, the two groups were homogeneous in all demographic and clinical characteristics, except for age.

The 6MWT (primary outcome) increased from 48.60 ± 42.39 m (T1) to 139.24 ± 104.7 m (T2) in the EG and from 44.29 ± 59.15 m (T1) to 149.43 ± 130.15 m (T2) in the CG.

**Figure 2:**
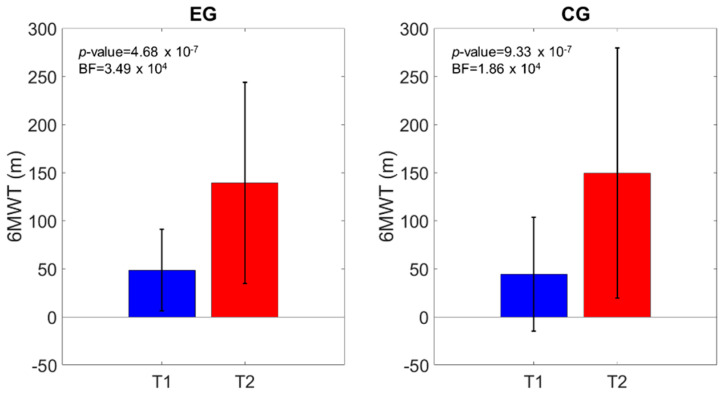
6MWT distance comparison of the scores at T1 and T2. The *p*-value and the Bayes Factor (BF) of the intragroup effects are shown.

The frequentist analysis revealed a significant time effect (*p*-value < 0.0001), while the time*group effect was not significant (*p*-value = 0.53). This was supported by the Bayesian analysis, which showed extreme (BF_01_ > 100 in both groups) relative evidence for the hypothesis of the difference between T1 and T2 (Table 2). The Bayesian model comparisons (Table 2) supported the model that included the factor of time when compared to the null model (BF_01_ = 1.669 × 10^10^). Figure 2 shows the improvement in the 6MWT in both the EG and the CG (*p*-value < 0.0001). Considering that the 6MWT MCID in subacute stroke subjects is 50.4 m [29], 55% and 57% of subjects exceeded such values in the EG and the CG, respectively. However, the statistical comparison of the number of subjects in the EG and the CG reaching (or not reaching) the 6MWT MCID threshold improvement revealed no statistical significance (*p*-value = 0.682).

**Figure 3:**
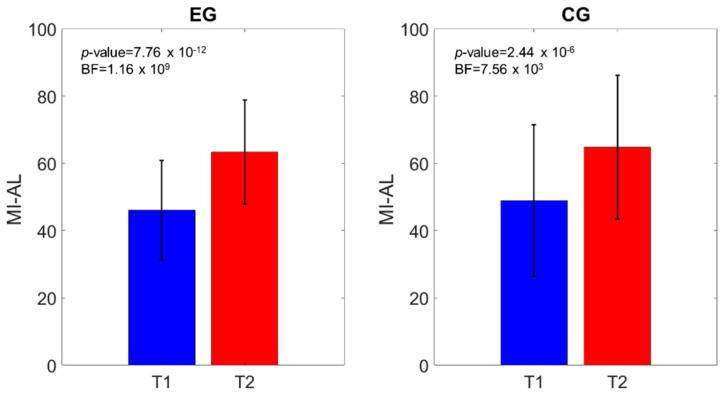
Motricity Index of the affected lower Limb (MI-AL) comparison of the scores at T1 and T2. The *p*-value and the BF of the intragroup effects are shown.

**Figure 4:**
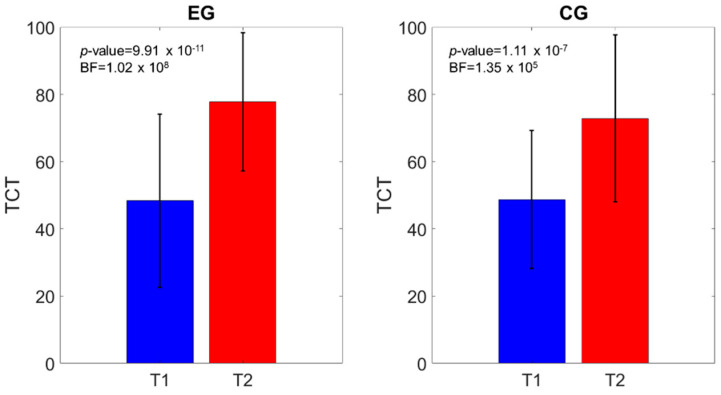
Trunk Control Test (TCT) comparison of the scores at T1 and T2. The *p*-value and the BF of the intragroup effects are shown.

**Figure 5:**
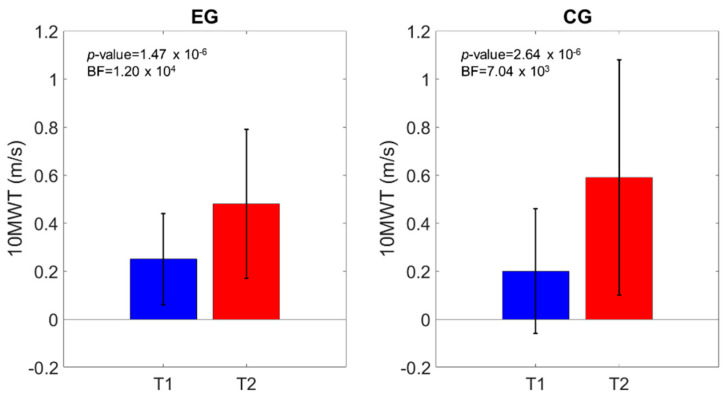
10MWT velocity comparison of the scores at T1 and T2. The *p*-value and the BF of the intragroup effects are shown.

**Figure 6:**
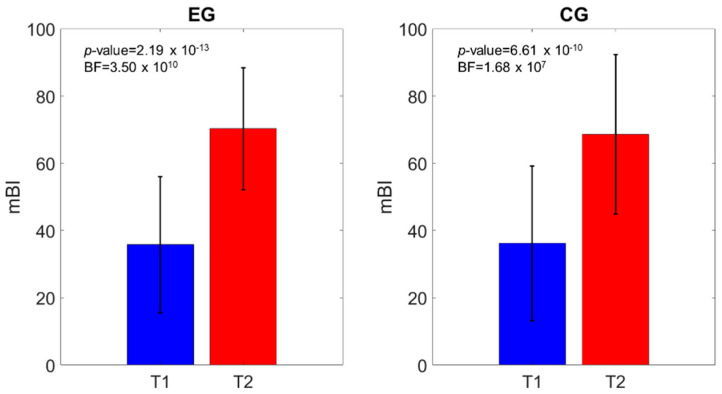
Modified Barthel Index (mBI) comparison of the scores at T1 and T2. The *p*-value and the BF of the intragroup effects are shown.

Similar outcomes were registered for the MI-AL (time effect: *p*-value < 0.0001; time*group effect: *p*-value = 0.64), TCT (time effect: *p*-value < 0.0001; time*group effect: *p*-value = 0.28), 10MWT (time effect: *p*-value < 0.0001; time*group effect: *p*-value = 0.05), and mBI (time effect: *p*-value < 0.0001; time*group effect: *p*-value = 0.66). Figure 3, Figure 4, Figure 5 and Figure 6 show that both the frequentist and Bayesian analyses revealed strong evidence in favor of improvement in all clinical outcomes at the end of treatment and in both groups. Moreover, the Bayesian repeated measures ANOVA (Table 2) evidenced a strong probability of the time model compared with the null one, thus supporting the hypothesis that both the traditional gait training and the o-RAGT improved body function and activity.

**Figure 7:**
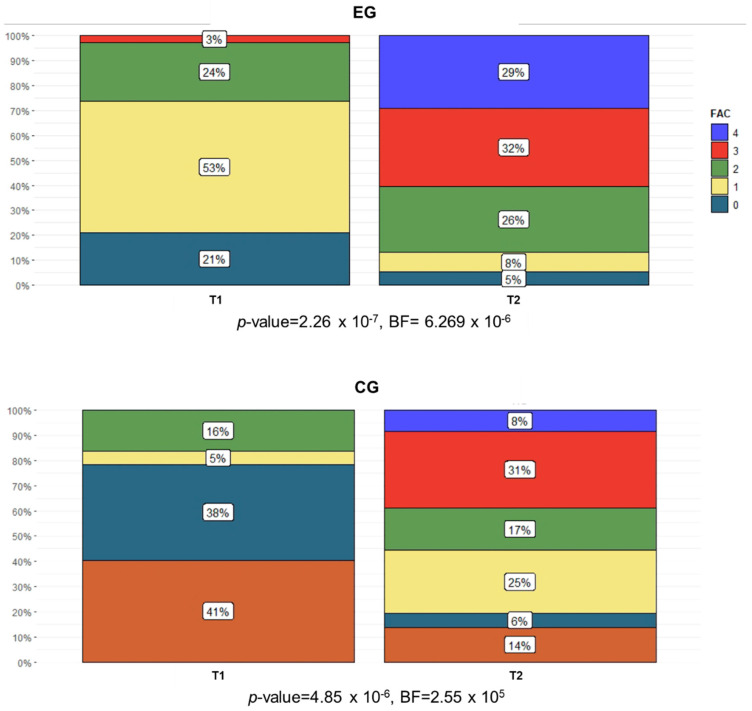
Percentage of subjects in each FAC category (0 = subject cannot walk; 1 = subject requires physical assistance from one person and contacts are continuous; 2 = subject requires physical assistance as in the previous category, but contact is intermittent or light; 3 = subject requires verbal supervision or standby help from one person without physical contact; 4 = subject can walk independently on level ground but requires help on stairs, slopes, or uneven surfaces; 5 = subject can walk independently anywhere) in both the EG and the CG at T1 and T2.

**Figure 8:**
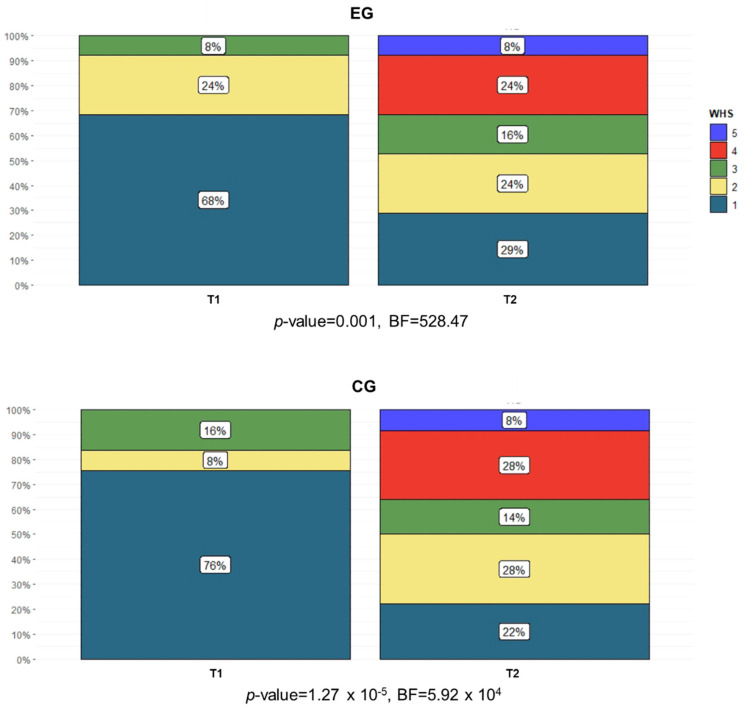
Percentage of subjects in each WHS category (1 = physiological gait; 2 = indoor gait with limitations; 3 = indoor gait without limitations; 4 = community ambulation with major limitations; 5 = community ambulation with some limitations; 6 = community ambulation without limitations) in both the EG and the CG at T1 and T2.

The obtained values of the categorical variables showed that, at the baseline, most of the subjects registered moderate spasticity (MAS-AL), medium-to-low functional ambulation ability (FAC), and limited abilities to walk in community ambulation (WHS). The chi-squared test of independence (Table 3) registered significant intergroup differences in the FAC and WHS at T1 and T2, respectively. Specifically, at the baseline, the subjects were non-functional ambulators or dependent ambulators who required assistance from another person. At the end of the treatments, 29% and 8% of subjects were independent ambulators who could walk freely only on level surfaces in the EG and the CG, respectively. Similarly, the WHS scores evidenced that the subjects were physiological and indoor walkers at the baseline, while at the end of treatment, they improved their community ambulation, with 8% of them classified as community walkers in both groups. The MAS-AL did not show any significant intragroup difference, while the FAC (Figure 7) and WHS (Figure 8) registered a significant number of subjects improving their functional and community ambulation in both groups. These results were confirmed by both the frequentist and Bayesian analyses.

The subject’s usability and acceptance of the WPE were measured with the questionnaire. The results showed that the subjects perceived the robotic treatment positively. Specifically, it was considered comfortable (5.78 ± 1.81), pleasant (5.91 ± 1.50), moderately painful (1.09 ± 2.09), and demanding (2.96 ± 2.10). Moreover, the subjects perceived the WPE as useful (6.22 ± 1.13), they would recommend it (6.35 ± 1.07), and they would like to do further o-RAGT in the near future (5.57 ± 2.23).

## 4. Discussion

This study enrolled subacute stroke subjects in a multicenter, randomized, controlled trial for investigating the efficacy of o-RAGT compared with conventional gait training. No adverse events were evidenced during the treatments, as reported in preliminary studies carried out with the same WPE [20,21,22]. The clinical experience gained during this study suggests that o-RAGT is a safe and intensive (i.e., high number of steps) form of gait training for stroke subjects, and it may reduce the therapist’s physical effort, in accordance with the literature on the topic [3,4,7].

The walking endurance (6MWT), which was the primary outcome of the study, increased at the end of treatment in both the EG and the CG. It registered a significant time effect in both the frequentist and Bayesian analyses, which strongly supports the model including the time effect. However, no group effect was obtained. This outcome is supported by the number of subjects who improved at the 6MWT more than the MCID, which was similar in both groups. These results are in accordance with recent preliminary studies on robot-assisted gait training in subacute stroke subjects [12,17,21,22,23].

The outcomes obtained in the body function and structure ICF domain revealed that the limb muscle strength (MI-AL) improved in both groups between T1 and T2, in accordance with the literature [3,4]. The Bayesian model, including the group factor, was not evident compared with the null model, thus indicating a similar improvement in the MI-AL in both groups. The spasticity (MAS-AL) was limited in both groups at the baseline, and these values did not increase at the end of the treatments. Therefore, it suggests that the o-RAGT did not increase spasticity in the subacute phase, in line with studies which employed robots for rehabilitation [13,16,20,21,22,36].

For the activity ICF domain, the FAC at the baseline revealed that 23% of the subjects were not able to walk (FAC = 0), and the majority of them (75% in the EG and 67% in the CG) regained the capacity to walk after the treatments. A significant number of subjects improving their functional ambulation was registered in both groups. A similar result was found in other studies [16,18,19,22]. The maximum velocity walked on a pathway 10 m long (10MWT) registered a significant time effect and no group effect, similar to the 6MWT. The results were in accordance with the study by Calabrò et al. [13].

For the participation ICF domain, community ambulation (WHS) measured at the baseline revealed limited abilities in all recruited subjects. At T1, the WHS was assessed by considering subjects in hospital areas (e.g., garden, cafeteria, and chapel) [37]. At the end of the treatments, both groups improved their walking in the community. The results are consistent with outcomes reported in similar studies on o-RAGT [15,20,21,22].

Cognitive and behavioral problems [38] are frequent complications after a stroke. As WPEs are characterized by close cognitive and physical interaction with the subject [6], the o-RAGT can be seen not only as physical training of the walking task, but also as cognitive training of intention. Specifically, the intention to move is a cognitive process that includes comprehension, visual perception, attention, memory, and motor control. In the context of motor control, this cognitive process leads to the planning and execution of the motor action. Therefore, the continuous interaction between the subject and the WPE directly stimulates the phenomena involved in the cognitive process [39]. In our study, we assessed the quality of this continuous interaction with a questionnaire on the subject’s experience. The acceptance of o-RAGT revealed that subacute stroke subjects perceived the practice with the WPE positively and found it useful.

In conclusion, our results showed that o-RAGT is feasible and tolerated well by subacute stroke subjects. Moreover, the WPE allows unable walking subjects to conduct an early and intensive overground gait experience. The effects of the treatment are comparable to the ones obtained with conventional gait training.

The main limitation of the study is the number of recruited subjects. A considerable number of subacute stroke subjects would be suggested for confirming the results obtained in the present study. However, considering the limitation of the scientific literature on the topic [17], to the best of our knowledge, this RCT is one of the first attempts to assess the efficacy of o-RAGT in subacute stroke subjects. Inspired by the literature on treadmill-based RAGT [16] and by the encouraging preliminary results comparing different devices [16,40], future large RCTs are needed to compare the effects of overground RAGT to RAGT with body weight support. Certainly, the research agenda should include the analysis of gait biomechanics and neurophysiological measurements [41] in order to better understand the underlying effects of o-RAGT in stroke subjects and to find clinical and demographic characteristics that could make a subject appropriate to robotic gait training.

## 5. Conclusions

The overground WPE was tolerated well and increased endurance in a similar way to the control group. Unable walking subjects who used the WPE could conduct intensive overground gait training and move the lower limbs through a pre-programmed physiological gait pattern. Moreover, the subject–robot cognitive interaction allowed the subject to understand the potential of the robot, fully rely on the robot, and control the robot’s movements. The clinical outcomes of o-RAGT (in combination with daily conventional therapy) were similar to those of conventional gait training for subacute stroke subjects who were able to stand upright. The results obtained in this RCT on subacute stroke subjects are encouraging and suggest future studies on o-RAGT, assessing clinical, biomechanical, and cognitive outcomes.

## Figures and Tables

**Table 1:** Demographic and clinical characteristics of the sample at T1.

	EG (*N* = 38)	CG (*N* = 37)	*p*-Value	BF_01_
Age (years)	62.13 ± 8.75	68.24 ± 8.58	0.003 ^1^	0.08
Gender (female or male)	17(45%)/21(55%)	19(51%)/21(49%)	0.896 ^2^	1.82
Etiology (ischemic or hemorrhagic)	30(79%)/8(21%)	33(89%)/4(11%)	0.291 ^2^	1.42
Affected side (right or left)	19(50%)/19(50%)	11(30%)/26(70%)	0.073 ^2^	0.39
Delay since stroke (days)	35.68 ± 10.70	34.14 ± 16.07	0.626 ^1^	3.78
6MWT (m)	48.60 ± 42.39	44.29 ± 59.15	0.906 ^1^	4.18
MI-AL	46.08 ± 14.80	48.97 ± 22.58	0.434 ^1^	3.19
TCT	48.37 ± 25.78	48.75 ± 20.54	0.849 ^1^	4.14
10MWT (m/s)	0.25 ± 0.19	0.20 ± 0.26	0.590 ^1^	3.67
mBI	35.78 ± 20.24	36.20 ± 22.98	0.891 ^1^	3.97

Mean ± standard deviation; *N* (%); EG = Experimental Group; CG = Control Group; 6MWT = distance of the 6 Minutes Walking Test; MI-AL = Motricity Index of the Affected lower Limb; TCT = Trunk Control Test; 10MWT = velocity of the 10 Meters Walking Test; mBI = modified Barthel Index; ^1^ *p*-value of the frequentist *t*-test; ^2^ *p*-value of the chi-squared test of independence; BF_01_ = Bayes factor.

**Table 2:** Repeated measurement ANalysis Of VAriance (ANOVAs) of the outcome measures using Bayesian statistics.

**6MWT**					
**Model Comparison**	**P (M)**	**P (M|Data)**	**BF_M_**	**BF_10_**	**Error %**
Null model (including age, subject)	0.200	4.346 × 10^−11^	1.738 × 10^−10^	1.000	-
Group	0.200	1.077 × 10^−11^	4.306 × 10^−11^	0.248	5.499
Time	0.200	0.725	10.571	1.669 × 10^10^	2.109
Group + Time	0.200	0.212	1.075	4.874 × 10^9^	3.048
Group + Time + Group ✻ Time	0.200	0.063	0.268	1.443 × 10^9^	4.104
**Effects**	**P (incl)**	**P (incl|Data)**	**BF_Inclusion_**		
Group	0.600	0.275	0.252	-	-
Time	0.600	1.000	1.230 × 10^10^	-	-
Group ✻ Time	0.200	0.063	0.268	-	-
**MI-AL**					
**Model Comparison**	**P (M)**	**P (M|Data)**	**BF_M_**	**BF_10_**	**Error %**
Null model (including age, subject)	0.200	1.434 × 10^−13^	5.736 × 10^−13^	1.000	-
Group	0.200	4.132 × 10^−14^	1.653 × 10^−13^	0.288	1.873
Time	0.200	0.656	7.619	4.573 × 10^12^	1.722
Group + Time	0.200	0.279	1.547	1.945 × 10^12^	3.550
Group + Time + Group ✻ Time	0.200	0.065	0.280	4.559 × 10^12^	4.303
**Effects**	**P (incl)**	**P (incl|Data)**	**BF_Inclusion_**		
Group	0.600	0.344	0.350	-	-
Time	0.600	1.000	3.609 × 10^12^	-	-
Group ✻ Time	0.200	0.065	0.280	-	-
**TCT**					
**Model Comparison**	**P (M)**	**P (M|Data)**	**BF_M_**	**BF_10_**	**Error %**
Null model (including age, subject)	0.200	4.045 × 10^−15^	1.618 × 10^−14^	1.000	-
Group	0.200	1.003 × 10^−15^	4.012 × 10^−15^	0.248	2.076
Time	0.200	0.658	7.709	1.628 × 10^14^	2.044
Group + Time	0.200	0.247	1.310	6.098 × 10^13^	4.351
Group + Time + Group ✻ Time	0.200	0.095	0.420	2.348 × 10^13^	3.005
**Effects**	**P (incl)**	**P (incl|Data)**	**BF_Inclusion_**		
Group	0.600	0.342	0.346	-	-
Time	0.600	1.000	1.334 × 10^14^	-	-
Group ✻ Time	0.200	0.095	0.420	-	-
**10MWT**					
**Model Comparison**	**P (M)**	**P (M|Data)**	**BF_M_**	**BF_10_**	**Error %**
Null model (including age, subject)	0.200	2.295 × 10^−9^	9.179 × 10^−9^	1.000	-
Group	0.200	6.818 × 10^−10^	2.727 × 10^−9^	0.297	3.271
Time	0.200	0.569	5.285	2.480 × 10^8^	2.906
Group + Time	0.200	0.186	0.914	8.103e × 10^7^	2.771
Group + Time + Group ✻ Time	0.200	0.245	1.297	1.067 x 10^8^	3.498
**Effects**	**P (incl)**	**P (incl|Data)**	**BF_Inclusion_**		
Group	0.600	0.431	0.505	-	-
Time	0.600	1.000	2.240 × 10^8^	-	-
Group ✻ Time	0.200	0.245	1.297	-	-
**mBI**					
**Model Comparison**	**P (M)**	**P (M|Data)**	**BF_M_**	**BF_10_**	**Error %**
Null model (including age, subject)	0.200	7.951 × 10^−21^	3.180 × 10^−20^	1.000	-
Group	0.200	1.887 × 10^−21^	7.548 × 10^−21^	0.237	2.206
Time	0.200	0.683	8.618	8.590 × 10^19^	1.547
Group + Time	0.200	0.251	1.338	3.152 × 10^19^	2.263
Group + Time + Group ✻ Time	0.200	0.066	0.285	8.353 × 10^18^	2.239
**Effects**	**P (incl)**	**P (incl|Data)**	**BF_Inclusion_**		
Group	0.600	0.317	0.309	-	-
Time	0.600	1.000	∞	-	-
Group ✻ Time	0.200	0.066	0.285	-	-

P (M) = prior model probability; P (M|data) = posterior model probability; BF_M_ = change from prior to posterior model odds; BF_10_ = Bayes factor for each model against the null model; P (incl) = prior inclusion probability; P (incl|data) = posterior inclusion probability; BF_Inclusion_ = inclusion Bayes factor. ✻ means interaction.

**Table 3:** Results of the chi-squared test between the Modified Ashworth Scale of the Affected lower Limb (MAS-AL), Functional Ambulation Classification (FAC), and Walking Handicap Scale (WHS) classes and the Experimental Group (EG) and Control Group (CG) samples.

	T1			T2		
	EG (*N* = 38)	CG (*N* = 37)	*p*-Value	EG (*N* = 38)	CG (*N* = 37)	*p*-Value
**MAS-AL**						
0.0	15	19	0.154	12	19	0.239
1.0	5	10		8	9	
1.5	1	0		1	0	
2.0	7	4		7	6	
2.5	1	1		2	0	
3.0	2	2		4	2	
3.5	0	0		3	0	
4.0	6	0		0	1	
4.5	1	0		1	0	
5.0	0	1		0	0	
5.5	6	0		0	0	
**FAC**						
0	8	15	0.010	2	5	0.261
1	20	14		3	2	
2	9	2		10	9	
3	1	6		12	6	
4	0	0		11	11	
5	0	0		0	3	
**WHS**						
1	26	28	0.961	11	8	0.001
2	9	3		9	10	
3	3	6		6	5	
4	0	0		9	10	
5	0	0		3	3	

EG = experimental group; CG = control group; MAS-AL = modified Ashworth scale of the affected lower limb; FAC = Functional Ambulation Classification; WHS = Walking Handicap Scale. The descriptive secondary analysis revealed that 52 (69%) subjects were able to walk at T1, with 30 in the EG and 22 in the CG. At T2, the able walking subject subgroups increased the distance walked in 6 min time by 99.5 m in the EG and by 109.6 m in the CG. Twenty-three subjects (8 in EG and 15 in the CG) were not able to walk at T1. Six subjects (75%) from the EG regained the capacity to walk after the overground Robot-Assisted Gait Training (o-RAGT), showing a 6 Minutes Walking Test (6MWT) = 76.50 ± 61.13 m and 10 Meters Walking Test (10MWT) = 0.29 ± 0.19 m/s at T2. Ten subjects (67%) from the CG were able to walk after the conventional gait training with a 6MWT = 127.40 ± 90.39 m and 10MWT = 0.51 ± 0.41 m/s.

## Data Availability

The data presented in this study are available on request from the corresponding author.
